# Effectiveness of implemented global dietary interventions: a scoping review of fiscal policies

**DOI:** 10.1186/s12889-024-19988-4

**Published:** 2024-09-19

**Authors:** Wisdom Dogbe, Faical Akaichi, Vanessa Rungapamestry, Cesar Revoredo-Giha

**Affiliations:** 1 The Rowett Institute, University of Aberdeen, Foresterhill, Aberdeen, AB25 2ZD UK; 2 Scotland’s Rural College (SRUC), Peter Wilson Building, King’s Buildings, West Mains Road, Edinburgh, EH9 3JG UK

**Keywords:** Fiscal policies, Sweetened beverages, Tax, Subsidy, Energy-dense food

## Abstract

**Background:**

Although the World Health Organisation (WHO) has proposed the use of fiscal policies to mitigate consumption externalities such as overweight and obesity-related diseases, very little is known about the impacts of the different types and framing of national and/or regional fiscal policies that have been implemented over the years. There is the need to provide up-to-date evidence on the impact of fiscal policies that have been enacted and implemented across the globe.

**Methods:**

We conducted a scoping review of all implemented government fiscal policies in the food and drinks sector to identify the different types of fiscal policies that exist and the scope of their impact on consumers as well as the food environment. Electronic databases such as the Web of Science and Google Scholar were used to search for appropriate literature on the topic. A total of 4,191 articles were retrieved and 127 were synthesized and charted for emerging themes.

**Results:**

The results from this review were synthesized in MS Excel following Arksey & O’Malley (2005). Emerging themes were identified across different countries/settings for synthesis. The results confirms that fiscal policies improve consumers’ health; increase the prices of foods that are high in fats, sugar, and salt; increase government revenue; and shift consumption and purchases towards healthier and untaxed foods.

**Conclusion:**

Governments already have the optimum tool required to effect changes in consumer behaviour and the food environment.

**Supplementary Information:**

The online version contains supplementary material available at 10.1186/s12889-024-19988-4.

## Introduction

Scotland is known for eating too much of the wrong things [1]. The food environment is populated with inexpensive salt, fat, and sugary foods. Poor dietary choices have resulted in an increased risk of obesity-related diseases such as hypertension, cardiovascular diseases, type 2 diabetes and certain types of cancers [2–4]. Statistically, in 2021, a total of 3.1 million people in the UK were registered to have diabetes, 700,000 more than in 2010 [5]. A switch from the consumption of discretionary foods^1^—high fat, salt, and sugar foods—to healthy diets high in fruit and vegetables, oil-rich, fibre and whole grains—is required to reduce the burden of diseases in Scotland. However, poor dietary choices are known to persist among people living in the most deprived areas [1].

Food Standard Scotland (FSS) data show that currently, the average person in Scotland consumes 15.1% of energy from saturated fat, which is 4% higher than the recommended percentage. In addition, 14.4% of the energy is derived from sugar, which is 9.4% above the recommended level. The average salt intake is 7.8 g, which is 1.8 g greater than the recommended intake [1].

A 2018 FSS report suggested that 65% of Scotland’s population is either overweight or obese [1]. In 2019, approximately 29% of Scottish adults were classified as obese, ranging from 23% in the least deprived areas to 36% in the most deprived areas. The prevalence of obesity-related NCD has slowly increased since 2014. Estimates show that the rate of obesity-related noncommunicable diseases (NCD) deaths could increase by 10%, from 56 per 100,000 to 62 per 100,000 [6]. In addition, 10% and 20% of five-year-olds and 11-year-olds, respectively, are obese [5], indicating a gloomy health outlook for Scotland.

In addition, a total of 6,697 and 2,181 deaths due to coronary heart disease (CHD) and stroke, respectively, were recorded in 2016. Sadly, 31% of children experience dental decay, while 29% of the population has high blood pressure [1]. NCD such as heart disease, cancer, diabetes, stroke, and liver and lung diseases were the leading causes of death in Scotland, accounting for almost 2/3 of all deaths in 2020. However, studies have shown that 1 in 5 of these deaths could be prevented through public health actions involving unhealthy food and drinks as well as tobacco and alcohol. Estimates suggest that poor health and disability caused by tobacco, alcohol and unhealthy food and drink costs the Scottish economy between £5.6 and £9.3 billion every year [7–9]. These statistics demand that policymakers engage with the food system to address these problems.

A recent survey by FSS suggested that more than half of Scottish adults want to see the Scottish Government do more to improve health. The first step is to nudge consumers to reduce the number of discretionary foods consumed by at least half [1]. Suggestions for the government to improve healthy choices include influencing marketing, price and promotion and the availability of unhealthy foods to the populace [10]. Price and promotions are the two dominant tools used by the food industry to drive the consumption of unhealthy products. According to The Food Foundation (2021), 46% of food and drink advertisements involve confectionery, sweet and savoury snacks and soft drinks, while only 2.5% involve fruits and vegetables.

Internationally, many countries and jurisdictions have introduced policies, programs, and guidelines to nudge consumers towards healthy eating. In the UK, the soft drink industry levy, five-a-day campaign, and the Eatwell Guide are the most prominent. Despite the implementation of these policies, the National Health Service is still overburdened by the cost of treating diet-related NCD. As a result, there is a high political interest in taxes and subsidies to improve diets and prevent the economic burden of diseases. Fiscal policies such as taxes come in different forms and sizes, including ad valorem taxes, value-added taxes (VATs), excise taxes, and import tariffs and taxes^2^. Theoretically, taxes (subsidies) create fiscal incentives for buyers to buy less (more) of affected foods, recalibrating overall diet quality [11]. Subsidising nutrient-rich foods^3^ is relevant because the poorest households in the UK would need to spend more than 70% of their disposable income on food to meet the UK’s Eatwell Guide [5]. Moreover, 10% of children live in households facing severe food insecurity, while 16% of UK adults skip meals due to a lack of money [5]. Ironically, unhealthy foods are three times cheaper than healthy foods.

The World Health Organisation strongly supports the use of fiscal measures to reduce the consumption of nutrient-poor, energy-dense foods [11, 12]. As a result, many countries and jurisdictions such as USA, Mexico, United Kingdom, Chile, Portugal, South Africa, Samoa, Bermuda, Ecuador, Ireland, Mauritius, Mexico, Norway, etc. have implemented fiscal policies to nudge consumers towards eating healthily. However, to our knowledge, there is no synthesis of worldwide studies assessing the impact of existing fiscal policies and drawing lessons that could help shape the food arena in Scotland and the UK. Previous literature reviews are based on simulation studies, including experimental and modelling studies. This scooping review goes beyond previous works by (1) presenting a comprehensive summary of all the fiscal policies implemented globally, (2) focusing on empirical studies based on implemented fiscal policies (excluding simulation studies), and (3) grouping the identified impact under broad themes relevant to policymakers. This review collates diverse research work from different jurisdictions under specific themes to help policy makers make informed decisions about the direction of impact.

The results from the current scoping review show that fiscal policies have significant impacts irrespective of the goal of the government. The positive aspects of fiscal policies include reducing the consumption of targeted foods, increasing the consumption of healthy untargeted foods, and increasing revenue to support government and health goals, i.e., reducing overweight and NCD. On the negative side, taxes increase the cost of consumption, especially for low-income households.

## Methods

### Literature search strategy

The following electronic databases were used to search for appropriate literature on the topic: PubMed, Academic Search Premier, Web of Science and Google Scholar. A keyword search strategy was developed and based on three main concepts using the search function “AND” to identify relevant articles: “tax/subsidy/fiscal”, “food/nutrition/diet/sugar-sweetened/energy-dense” and “policy/program”. The “OR” function was used to vary the keywords or concepts to expand the results. The search was implemented using (“tax” OR “subsidy” OR “fiscal”) AND (“food” OR “nutrition” OR “diet” OR “sugar-sweetened” OR “energy-dense”) AND (“policy”).

The inclusion criteria were restricted to studies related to fiscal policies that have been implemented and evaluated across various jurisdictions across the world irrespective of methodology or depth of analysis. The period during which the policy or program was implemented and whether it was ongoing or abolished were irrelevant^4^. However, since most fiscal policies on nutrition started in the 1980s, the search period started from 1980 to 2022. The goal is to identify fiscal policies that have been implemented to improve nutrition and/or health.

Studies that were not based on existing government policies were excluded from the analysis. Additionally, studies based on fiscal policies directed towards agriculture, inputs/fertiliser, trade, and farming were excluded from the final analysis. Studies that were not directed towards health or nutrition were excluded. Finally, simulation studies that were not based on existing government policies or programs were also excluded.

We followed the criteria suggested by Arksey & O’Malley [13] to refine the literature for inclusion and exclusion. Before the review, the primary author ensured that duplicate studies were excluded based on the titles of the studies. Examination of the remaining articles was based on their titles, followed by their abstracts and then the full paper. The references of the articles were screened to increase the number of articles included. All articles were independently reviewed by WD, FA, and where there is disagreement VR and CRG were consulted. The final articles included in the final review were charted by WD and refined by the remaining authors (FA, VR, CRG).

Data from the articles were charted using MS Excel following Arksey & O’Malley [13]. The information collected for further analysis included author(s), study country/location, setting intervention, measurable outcomes, effect on outcomes, year, data and method. Emerging themes were identified across different countries/settings for synthesis.

### Data abstraction and synthesis

We followed the work of [13, 14] by charting through the literature to synthesise studies relevant to the topic. The data from the studies were analysed using Microsoft Excel, and the characteristics of the studies considered included the name of the authors, the description of the intervention, the country and year the intervention was implemented, and the outcome of the study assessing the impact of the intervention. Outcomes from the various studies were coded, and emerging themes were identified for the results and discussion.

**Fig. 1:**
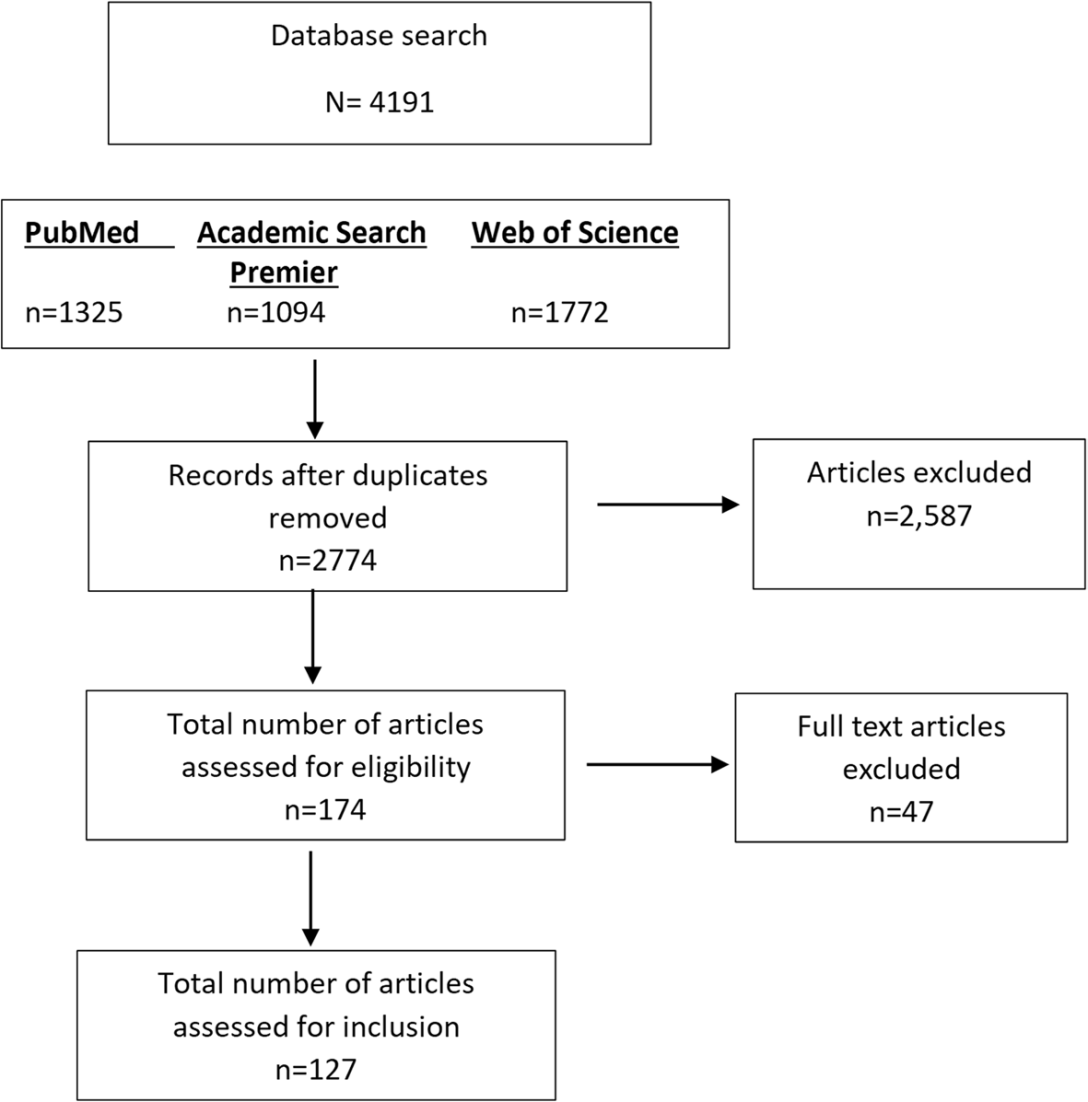
Flowchart of search results Source: Own computation based on literature search

## Results

### Search outcome

A total of 4,191 articles were retrieved from the three databases shown in Fig. 1. Approximately 2,587 duplicate articles were excluded from the total. Through manual searching, 5 articles were included in the review (mainly from Google Scholar). Table 1 shows the countries and the number/percentage of studies found; the USA had the highest number of studies (44), followed by Mexico (18), the United Kingdom (13), South Africa (5), Portugal (5) and Chile (5). Additionally, there were studies from Australia, Barbados, Bermuda, Canada, Denmark, Ecuador, France, French Polynesia, Hungary, Ireland, Mauritius, Navajo, Norway, the Philippines, Samoa, Saudi Arabia, Spain, Thailand, and Tonga. Tax policies had an impact on household purchases/retail sales, consumer welfare, government revenue, health, diet, and nutrition across 24 different jurisdictions.

**Table 1:** Countries and the number of studies considered for synthesis

Country	Number of Publications	Percentage publications
USA	44	34.6
Mexico	18	14.2
United Kingdom	13	10.2
Chile	5	3.9
Portugal	5	3.9
South Africa	5	3.9
Denmark	4	3.1
France	4	3.1
Hungary	4	3.1
Spain	4	3.1
Saudi Arabia	3	2.4
Barbados	2	1.6
Philippines	2	1.6
Thailand	2	1.6
Tonga	2	1.6
Australia	1	0.8
Canada	1	0.8
French Polynesia	1	0.8
Samoa	1	0.8
Bermuda	1	0.8
Ecuador	1	0.8
Ireland	1	0.8
Mauritius	1	0.8
Mexico	1	0.8
Norway	1	0.8

Source: Authors’ computation based on the literature search

### Theme 1: Tax policies may affect household purchases/consumption/sales

This theme considers the impact of taxes on household purchases or consumption and sales across different jurisdictions and policy scenarios. Table 2 shows that tax policies are effective in reducing household purchases and sales.

**Table 2:** Summary of results on the implications of taxes on consumption, purchases, sales or volume sold

Author	Country	Intervention	Effect on outcomes	Year Implemented
Alvarado M et al. (2019) [15]	Barbados	A 10% ad valorem (value-based) tax on SSBs	Purchases of taxed foods fell	2015
Caro JC et al. (2020) [16]	Chile	(1) SSB tax revised from 13–18% for SSBs with sugar greater than 6.25 g sugar/100 mL; (2) SSB tax revised from 13 − 10% for SSBs with sugar less than 6.25 g sugar/100 mL	Combined policies are more effective	2014
Caro JC et al. (2018) [17]	Chile		The impact of the policy was small	2014
Nakamura R et al. (2018) [18]	Chile		Reduction in soft drinks demand	2014
Smed S et al. (2016) [19]	Denmark	Tax 16 DKK/kg saturated fat (2.14€/kg) on foods with saturated fat above 2.3 g/100 g.	Saturated fat purchases fell	2011
Bødker M et al. (2015) [20]	Denmark		Saturated fat purchases fell	2011
Jensen et al. (2016) [21]	Denmark		Saturated fat purchases fell from minced beef and regular cream	2011
Kurz and König, (2021) [22]	France	An excise tax of 0.0716 Euro/Litre on the producer price of SSBs	Total SSB sales declined but soft drinks sales increased	2012
Capacci et al. (2019) [23]	France		Reduction in purchases of taxed beverages	2012
Thow AM et al. (2011) [24]	French Polynesia	SSBs with sugar less than 5 g sugar/100 ml; 20 CFP/L for 5 to 9.99 g sugar/100 ml; 30 (imp) or 40 (prod) CFP/L for 10 to 29.99 g sugar/100 ml; 45 (imp) or 60 (prod) CFP/L for 30 to 39.99 g sugar/100 ml; 60 (imp) or 85 (prod) CFP/L CFP/L for 40 g + sugar/100 ml+.	Revenue increased	2002
Bíró A (2015) [25]	Hungary	A 4-cent tax on foods high in salt, sugar or caffeine	Purchases reduced	2011
Kurz and König, (2021) [22]	Hungary		An overall increase in SSB sales	2011
Zámbó et al. (2020) [26]	Hungary		Consumption decreases with tax awareness	2011
Cawley, Daly, Thornton (2022) [27]	Mauritius	8 US cents per 100 g of sugar content	Reduced probability of SSB consumption among boys	2013
Colchero et al. (2016) [28]	Mexico	An excise tax of one peso ($0.008) per litre	Reduction in SSB sales, increase in water sales	2014
Sánchez-Romero LM et al. (2020) [29]	Mexico		Purchases of taxed beverages reduced	2014
Ng S et al. (2019) [30]	Mexico		Purchases of taxed beverages reduced	2014
Colchero MA et al. (2017) [31]	Mexico		Purchases of taxed beverages reduced	2014

The United Kingdom: United Kingdom Soft Drinks Industry Levy (SDIL) was announced in March 2016 and implemented in April 2018; it charges manufacturers and importers at £0.24 per litre for drinks with over 8 g of sugar per 100 mL (high levy category), £0.18 per litre for drinks with 5 to 8 g of sugar per 100 mL (low levy category), and no charge for drinks with less than 5 g of sugar per 100 mL (no levy category). Scarborough et al. [32] studied the impact of the announcement of the SDIL and found that the number of drinks in the high levy category fell by 3% when the SDIL was announced. Rogers et al. [33] found that the volume of all soft drinks purchased combined increased by 2.6% per household per week a year after the implementation of the tax. On the positive side, the amount of sugar consumed from soft drinks decreased by 2.7% per household per week over the same period. Dickson et al. [34] reported that the reformulation of the SDIL led to a 6,600 calories per year per capita reduction in soft drinks. Bandy et al. [35] reported that the volume of sugar sold per capita per day from soft drinks declined by 30% or 4.6 g per capita per day. In addition, the weight means sugar content of soft drinks decreased from 4.4 g/100 in 2015 to 2.9 g/100 in 2018. Sales of soft drinks subjected to the levy fell by 50%, while those exempted from the levy rose by 40%. Rogers et al. [36] found evidence of a small increase in sugar purchased from all drinks compared to before the announcement of the levy. Pell et al. [37] reported that one year after implementation, the volume of drinks purchased did not change, but sugar purchases declined by 9.8%. Dogbe and Revoredo-Giha [38], considering a tax pass-through of 50%, found that levies reduced annual volume purchases and sugar by 1.4% and 3.9%, respectively. Law et al. [39] found that the announcement of SDIL had a significant negative impact on the turnover of manufacturers; however, this was not carried out postimplementation.

Barbados: In 2015, the government of Barbados implemented a 10% ad valorem tax on SSBs. Alvarado et al. [15] estimated the impact of the policy on SSB purchases using electronic point-of-sale data. The authors applied an interrupted time series (ITS) design to assess grocery store SSB and non-SSB sales from January 2013 to October 2016. The authors found that sales for taxed SSBs decreased by 4.3%, while non-SSB sales increased by 5.2%.

Bermuda: Bermuda implemented a discretionary food tax based on import tariff changes on retail prices and sales of SSBs and tariff reductions for selected fruits and vegetables. The first country to implement both tax and subsidy policies concurrently. Assessing the implications of both policies, Segal et al. [40] found that the market share of SSBs decreased by 8% due to the tax; however, the subsidy policy had no significant effect on sales.

Chile: The Chilean government revised (increased) its SSB tax from 13 to 18% for SSBs with sugar greater than 6.25 g/100 mL and revised (decreased) the SSB tax from 13 to 10% for SSBs with sugar less than 6.25 g/100 mL in 2014. Caro et al. [16] assessed the implications of these changes in Chile using the Chilean Household Budget Survey. The authors found evidence of substitution for cheaper SSBs and a reduction in the average household’s sweetened beverage purchases of 0.9 L per month. Caro et al. [17] also assessed the implications of tax revisions for SSB purchases in Chile and reported that households decreased their monthly per capita purchases of SSBs with a sugar content greater than 6.25 g/100 mL by 3.4% by volume (4% by calories). However, the purchase of SSBs with less than 6.25 g of sugar/100 mL increased by 10.7%. Nakamura et al. [18] also used a fixed effect model to assess the implications of the Chilean SSB tax increase from 13 to 18% for SSBs with a sugar content greater than 6.25 g. The authors found a highly significant decrease in the monthly purchased volume of the taxed drinks by 21.6%.

Denmark: In 2011, the Danish government imposed a tax of 16 DKK/kg (2.14 €/kg) on foods with saturated fat above 2.3 g/100 g. Smed et al. [19] assessed the effect of this tax on food and nutrient intake in Denmark. According to the study, the tax resulted in a 4% decrease in saturated fat purchases. Bødker et al. [20] assessed the implications of the policy for health and consumption and concluded that the total sale of foodstuffs decreased by 0.9%. Another study by Jensen et al. [21] investigated the effects of the tax on meat and dairy demand. The authors found that the tax induced a total decrease of 4–6% in saturated fat intake from minced beef and regular cream but had no effect on the intake of sour cream. Finally, Jensen and Smed [41] assessed the short-term effects of the Danish fat tax on consumption, substitution patterns and consumer prices of fat and found that the level of consumption of fat decreased by 10–15%. In addition, they found that the purchase of butter, margarine, blends and oil decreased by approximately 10%.

Ecuador: Ecuador implemented a volumetric tax of 18 cents per Liter on sugary drinks with more than 25 g of sugar per Liter in 2016. Comparing the tax to a 20% ad-valorem tax, Segovia et al. [42] concluded that the tax imposed by the Ecuadorian government was less effective than the simulated ad-valorem tax.

France: In January 2012, the French soda tax was introduced and set to €0.0716 per liter on the producer price of SSBs. It is applied to all sweetened drinks, including sugar substitutes used in diet drinks, and is paid for by manufacturers, processors and importers [23]. The authors estimated the impact of the French soda tax on both purchases and prices using a difference-in-differences approach. The results indicate that a small reduction in soft drink purchases (approximately half a liter per capita per year) could be due to the low tax rate. Assessing the effect of the same policy, Kurz and König [22] found a slight decrease in SSB sales but an overall increase in soft drink sales. The two studies suggest that the French soda tax had a marginal impact on both purchases and sales.

Hungary: Hungary imposed a 4-cent tax public health product (PHPT) on foods high in salt, sugar, or caffeine in 2011. The objective was to promote healthier eating habits through reformulation and to increase revenues for public health. Assessing the effectiveness of the tax, Zámbó et al. [26] found that the consumption of taxed products increased in all categories (except for salty condiments) between 2013 and 2018. Bíró [25] assessed the effectiveness of the tax on the consumption of processed and unprocessed foods before and after the tax came into effect. The results from the study suggest that the consumption (in terms of quantities) of processed foods decreased by 3.4% due to the tax. Martos et al. [43] also found that the policy reduced the consumption of targeted taxed foods both in the short and long run. Kurz and König [22] assessed and compared the impact of the soda tax implemented in France and Hungary. The authors found a slight decrease in SSB sales after tax implementation, but overall soft drink sales increased in France. For Hungary, there was only a short-term decrease in SSB sales, which disappeared after 2 years, leading to an overall increase in SSB sales. The authors concluded that the tax had a short-term impact in Hungary but had no effect on soft drinks in France.

Ireland: Briggs et al. [44] assessed the potential health impact of a proposed 10% tax on SSBs in Ireland. The authors found that the proposed tax could reduce average energy intake by 2.1 kcal per person per day and reduce the percentage of the obese population by 1.3%.

Mauritius: In January 2013, the government of Mauritius imposed a tax on SSBs based on their sugar content. The tax applied to both locally manufactured and imported drinks was equivalent to 8 US cents per 100 g of sugar content. Cawley et al. [27] assessed the implications of the policy on youth consumption and body mass index using a difference-in-differences model. There was no evidence of an effect of the tax on SSB consumption for the full sample of youth, but subgroup analyses indicated that the tax reduced the probability that boys would consume SSBs by 9.1% points (11%).

Mexico: In 2014, the Mexican government implemented an excise tax of one peso ($0.008) per litre (equivalent to a 10% price increase) on SSBs except for medical beverage products. The tax was implemented by the Mexican Congress as an initiative to limit Mexico’s obesity epidemic. Colchero et al. [45] assessed the impact of the tax on SSB and water purchases across different locations, household types and income levels. Their results suggest that purchases of SSBs decreased by 6.3% in 2014 compared with the trend from 2008 to 2012. Additionally, water purchases increased by 16.2% during the same period. Colchero et al. [31] again estimated how consumers responded to the Mexican beverage tax two years after it was implemented. The results from the study revealed that purchases of taxed beverages decreased by 5.5% in 2014 and 9.7% in 2015 compared to purchases in 2012-13. Colchero et al. [28] assessed the impact of the tax on beverage sales before and after the implementation of the policy. The authors found a decrease of 7.3% in per capita sales of SSB and an increase of 5.2% in per capita sales of plain water in 2014–2015 compared to the pretax period (2007–2013). Ng et al. [30] assessed how highly SSB purchasers responded to the excise tax. The authors found that SSB purchasers had the largest absolute and relative reductions in taxed beverages and increased their purchases of untaxed beverages. Colchero et al. [46] estimated the impact of the tax on purchases of SSBs from retail stores one year after implementation. The results from the study suggest that beverage purchases decreased by 6% in 2014 compared with 2012 at a decreasing rate of up to 12%. Sánchez-Romero et al. [29] assessed the association between SSB tax and soft drink consumption among adults in Mexico using an open cohort longitudinal analysis of health workers. The authors compared four categories of consumers: non, high-, low- and medium-level consumers. The results from the study showed that the proportion of medium and high consumers of soft drinks decreased by 7% after the tax came into effect. In addition, the percentage of non-consumers of soft drinks increased by 4% (from 10 to 14%). Finally, Pedraza et al. [47] studied the effect of the SSB tax on the caloric and sugar content of beverages bought in different stores in Mexico. They found that the volume of SSBs purchased declined by 49 ml and 30 ml in 2014 and 2015, respectively.

The Mexican government also imposed an 8% tax on nonessential energy-dense foods with an energy density of 275 kcal/100 g or more in the same year the SSB tax was implemented. Batis et al. [48] assessed the effect of the tax on both taxed and untaxed packaged foods through an observational study. The results showed that purchases of taxed packaged foods were reduced by 5.1% per person per month. However, purchases of untaxed packaged foods remained the same. Taillie et al. [49] also assessed the impact of the nonessential energy-dense tax two years after its implementation by comparing the impact on high and low purchasers before and after the implementation of the tax. The tax was sustainable; decreases in purchases for taxed foods increased from 4.8% in the first year to 7.4% in the second year. Hernández-F et al. [50] also assessed the effect of the energy-dense tax on the purchases of energy-dense nutrient-poor foods a year after the policy was implemented. The results from the study showed that the purchases of energy-dense nutrient-poor foods decreased by an average of 5.3% in 2014–2016 compared with purchases made in 2008–2012. Focusing on snacks, Aguilera Aburto et al. [51] showed that the Mexican energy-dense tax resulted in a moderate reduction in the consumption of snacks.

Navajo Nation: In 2014, the Navajo Nation passed the Healthy Diné Nation Act (HDNA), which combined a 2% tax on foods of ‘minimal-to-no-nutritional value’ and a waiver of a 5% sales tax on healthy foods. George et al. [52] assessed the implications of the tax on the pricing and availability of unhealthy foods. The authors found that compared to border town stores, in 2019, the availability of fresh vegetables and fruits was greater in convenience stores in Navejo. Trujillo Lalla et al. [53] also assessed the impact of the tax on purchasing trends using a multiyear cross-sectional survey. They found trends towards reduced purchasing of SSBs due to the tax.

Norway: In January 2018, the Norwegian government increased taxes on chocolate and sugar products from 2.09 EUR per kg to 3.82 EUR per kg and taxes on non-alcoholic beverages from 0.35 EUR per litre to 0.49 EUR per litre. Assessing the implications of taxes on retail sales, Øvrebø et al. [54] did not detect any significant reductions in sales that coincided with the increase in taxes.

Pacific: Thow et al. [24] assessed the impact of the soda tax in the Pacific Empire, which consists of Fiji, Samoa, Nauru, and French Polynesia. In Samoa, survey data analysed by Keighley et al., [55] suggest that the number of servings of soda consumed by the Samoan population decreased slightly between 1991 and 2003, from approximately 2.5 to just over two servings per week.

Philippines: Additionally, in January 2018, the Philippines implemented a tax of 0.185 US dollars per litre on beverages containing locally sourced sweeteners and 0.37 US dollars per litre on beverages containing imported sweeteners. Assessments by Onagan et al. [56] showed that sales of sweetened beverages decreased significantly; the greatest decrease was 8.7% in convenience stores just a month after implementation.

Portugal: In February 2017, the Portuguese government implemented a tiered sugar-sweetened beverage tax on producers based on the amount of sugar contained in drinks. The rates are as follows: 1 euro cent per litre for drinks with less than 25 g of sugar per litre; 6 cents for drinks with 25–49.99 g of sugar per litre; 8 cents for drinks with 50–79.99 g of sugar per litre; and 20 cents for drinks with 80 g or more of sugar per litre. The goal was to incentivise firms to reformulate towards lower sugar content. Goncalves et al. [57] reported a significant decrease in the domestic sales of SSBs following the implementation of the policy. Goiana-da-Silva, Nunes, et al. [58] also found a 15% decline in the total volume of sugar consumed from all ranges of beverages covered by the tax. In addition, they estimate a decrease of 4.3% in sales. Goiana-da-Silva, Cruz-e-Silva, et al. [59] estimated a 7% reduction in sales and an 11% reduction in total energy intake from sweetened beverage consumption as a result of reformulation. Goiana-da-Silva et al. [60] reported a reduction of 6.6 million litres of SSBs sold per year due to the tax. In addition, the average energy density of the SSBs decreased by 3.1 kcal/100 ml as a result of product reformulation. In contrast, Gonçalves and Pereira dos Santos [61] found no impact of the consumption tax, except for low-sugar drinks.

Saudi Arabia: Saudi Arabia imposed a 50% excise on soft drinks and a 120% excise on energy drinks, which came into effect in 2017. Alhareky et al. [62] assessed the impact of the tax on SSB consumption among Saudi school children. The authors found that energy drink consumption declined by 8%, but soft drink consumption increased by 2% after tax implementation. However, Alsukait et al. [63] estimated a 35% reduction in the volume sales of soft drinks relative to other Araba Gulf states. Furthermore, Megally and Al-Jawaldeh [64] estimated a 57.64% decrease in the sales volume of soft drinks from 2010 to 2017 following the implementation of the policy.

South Africa: Last, in April 2018, the South African government implemented a health promotion levy (HPL) payable by producers and importers of sugary beverages at a rate of 2.1 cents per gram of total sugar over 4 g per 100 mL. Bercholz et al. [65] estimated a 4.9 gram per capita per day reduction in sugar purchases from SSBs following the announcement of the tax. Another study by Koen et al. [66] revealed that self-reported consumption of SSBs decreased by 7.7% after the HPL was enacted. Finally, Essman et al. [67] assessed the implications of the tax and showed that sugar intake decreased significantly from 28.8 g/capita/day pretax to 19.8 g/capita/day post-tax implementation. In addition, the volume intake decreased from 315 ml/capita/day pretax to 198 ml/capita/day post-tax.

Spain - Catalonia: In May 2017, Catalonia, a state in Spain, implemented a tax of 0.08€ per Liter on beverages containing between 5 and 8 g per 100 ml and 0.12€ per Liter on beverages containing more than 8 g per 100 ml. Assessing the implications of the tax, Fichera et al. [68] found a 2.2% reduction in purchases from beverages. Royo-Bordonada et al. [69] assessed the impact of the tax on young people living in poorer neighbourhoods in Catalonia using Madrid as a control group. The authors found a 39% reduction in the prevalence of regular consumers of taxed beverages. However, the prevalence of consumers of nontaxed beverages remained the same after the tax. Assessing the impact of the tax on SSB sales, Vall Castelló and Lopez Casasnovas [70] estimated a reduction of 7.7%. Focusing on the impact of the tax on Coca-Cola, Puig-Codina et al. [71] found that the policy significantly reduced the volume of purchases (12.1%) and penetration rates (1.27%) of regular cola. However, the volume of purchases and penetration of diet cola increased by 17% and 1.65%, respectively.

Tonga, Oceania: In August 2013, Tonga’s 15% import tariff on SBs was replaced with an excise tax of T$0.50/L (US$0.28/L, 42% of import value) and subsequently doubled to T$1.00/L in July 2016 (63% of import value). The excise is applied to full sugar and artificially sweetened soft drinks, energy drinks, and other SBs. Water (sparkling or flat), juice (sweetened or unsweetened), powdered juice drinks, tea, coffee or hot chocolate were exempted from the tax. Teng et al. [72] assessed the implications of the tax and found significant decreases in all soft drink purchases. Teng et al. [73] also reported that the imports of sweetened beverages decreased by 10.4%, 30.3% and − 62.5% in 2013, 2016 and 2017, respectively, after tax imposition.

Thailand: In September 2017, Thailand also imposed a tax on SSBs according to their sugar content. The SSB products that contain less than 6 g of sugar per 100 mL are exempt from the tax, while those products containing 6 g or more of sugar per 100 mL are taxed at a higher rate. This is expected to increase every two years based on inflation rates. By assessing the impact of the tax policy on both taxed and nontaxed SSBs, Phulkerd et al. [74] found a significant reduction in taxed SSBs compared with nontaxed ones.

Berkeley, USA: In November 2014, the city of Berkeley passed a penny-per-ounce levy on SSBs, which included soda, energy, sports and fruit-flavoured drinks; sweetened water, coffee, and tea; and syrups used in the production of SSBs. Falbe et al. [75] assessed the impact of the tax on sugar-sweetened beverage consumption. The results from the study showed that the consumption of SSBs declined by 21% in Berkley but increased by 4% in Oakland and San Francisco. However, water consumption increased more in Berkley than in Oakland and San Francisco. Silver et al. [76] assessed the implications of the tax on sales and found that sales of SSBs in Berkley declined by 9.6% but increased in controlled cities by 6.9%. The authors did not find a significant difference in self-reported SSB intake before and after tax imposition. The study by Lee et al. [77] was conducted 3 years after the implementation of the Berkley SSB tax. The authors found that SSB consumption was reduced by 0.55 times per day, while water consumption increased by 1.02 times per day. In addition, the changes in SSB and water consumption in Berkley were significantly different from those in the neighbouring city, San Francisco, and Oakland comparison groups.

Cook County, USA: Cook County, Illinois, implemented an SSB tax of a 1.00-cent-per-ounce tax on the retail sale of sweetened beverages on August 2, 2017, and later repealed, effective November 30, 2017. Assessing the changes in beverage prices and volume following the implementation and repeal of the tax, Powell and Leider [78] found that in the 4 months that the Sweetened Beverage Tax was in place, the volume sold decreased while the tax was in place, but the sales volume returned to their pretax levels 8 months after the tax was repealed. Similarly, Powell, Leider, and Léger [79] assessed the impact of the Cook County SSB tax on the volume of SSB sold in the city and its border area. They estimate a 27% reduction in the volume of SSB sold. However, the impact differed between soda and energy drinks, between artificially sweetened beverages and SSBs, and between family-size and individual-size beverages.

Oakland, USA: Cawley et al. (2020) assessed the impact of the Oakland 1 cent per ounce SSB tax on prices, purchases and consumption by adults and children. Although not statistically significant, the tax decreased purchases by 11.33 ounces per shopping trip. However, the tax did not reduce the consumption of SSBs or added sugars for either adults or children. In contrast, Léger and Powell [80] reported that the volume of taxed beverages sold decreased by 14%, but 46% of this decrease was offset by an increase in cross-border purchases.

Seattle, USA: In January 2018, Seattle implemented a 1.75 cent per ounce Sweetened Beverage Tax (SBT) on SSBs with at least 40 calories per 12 ounces; milk, including flavoured/sweetened milk, as well as 100% juice, was exempted from the tax. Powell, Leider, and Oddo [81] evaluated changes in the grams of sugar sold after the implementation of the tax policy using a difference-in-differences analysis. The authors found a 23% (28%) decrease in sugar sold from taxed beverages (soda) from the pretax period to year 1 and year 2 post-tax implementation. Powell and Leider [82] assessed the impact of the tax on prices, volume sold and cross-border shopping. They found that the average volume of taxed beverages sold fell by 22%, 29% for larger families versus 10% for individual families. Oddo, Leider, and Powell [83] compared the sales of sweets and salty snacks in Seattle and Portland and reported that Seattle SBT increased the sales of sweets by 4% and 6%, respectively, a year and two years after implementation. However, there was no impact on the sales of salty snacks. Powell and Leider [84] reported a reduction of 22% in the volume of sugary drinks sold in Seattle following the implementation of the tax.

Philadelphia, USA: In 2017, Philadelphia imposed a beverage tax of $0.015/ounce on sugar (regular) and sugar substitute (diet) beverages. This was an excise tax paid by distributors. However, products containing more than 50% milk and 100% fruit drinks were exempted from the tax. Zhong et al. [85] assessed the immediate impact of the tax on the consumption of soda, fruit drinks, energy drinks, and bottled water. The authors found that the consumption of soda declined by 40% 2 months after the tax came into effect. Similarly, purchases of energy drinks were reduced by 64%, while bottled water purchases increased by 58%. Roberto et al. [86] further assessed the impact of taxes on beverage prices and sales at chain retailers in a large urban setting. They compared beverage prices and sales in Philadelphia with those in Baltimore, Maryland (a control city with a tax). The results showed that the total volume of sales of taxed beverages decreased by 1.3 billion dollars in Philadelphia; however, sales in Pennsylvania borders increased by 308.2 million ounces. A study by Bleich et al. [87] revealed that the purchase of taxed beverages declined by 6.1 fl. oz, corresponding to a 42% decline in Philadelphia compared with Baltimore (a controlled city). Edmondson et al. [88] also assessed the implications of tax SSBs among high school students. They found a reduction of 0.81 servings of soda per week 2 years after tax implementation. Longitudinal studies by Lawman et al. [89] did not find statistically significant changes in SSB purchases one year after the implementation of the Philadelphia beverage tax. However, an analysis excluding holiday purchasing or aggregating post-tax time revealed a reduction of between 4.9 and 12.5 ounces per day. Zhong et al. [90] assessed the effect of the tax on sugar-sweetened and diet beverage consumption and concluded that there was no overall impact on population-level consumption of sugar-sweetened or diet beverages or bottled water a year after the tax was implemented. Petimar et al. [91] found that the volume of sales of taxed beverages decreased by 35% (after adjusting for cross-border shopping) two years after the implementation of the tax. Bleich et al. [92] found larger declines in the volume of taxed beverages sold (5.76 ounces, or 38.9%) after tax implementation. After accounting for cross-border shopping to shops outside of Philadelphia, Seiler, Tuchman, and Yao [93] concluded that the tax led to a 22% reduction in sales. Additionally, Seiler, Tuchman, and Yao [93], analysed the impact of the Philadelphia SSB tax on calories and found that calories from beverages decreased by 16% after the implementation of the tax. According to Cawley et al. [94], the Philadelphia tax reduced the frequency of adults’ soda consumption by 31%, but no detectable impacts on children’s soda consumption were found. Grummon et al. [95] found a reduction in the purchases of taxed beverages following the implementation of the tax.

### Theme 2: Impact of taxes on prices/pass-through effect

A summary of the impact of tax policies on the prices of taxed beverages and pass-through effects is shown in Table 3. In summary, tax polices result in higher prices paid for by consumers at retail shops. However, the proportion of the tax paid for by consumers differs by jurisdiction, type of product, type of retail shop, etc.

**Table 3:** Summary of the effect of tax policies on price changes and the tax pass-through rate

Author	Country	Intervention	Effect on outcomes	Year
Alvarado M et al. (2017) [96]	Barbados	A 10% ad valorem (value-based) tax on SSBs	Prices of taxed beverages increased by 5.9%	2015
Caro JC et al. (2018) [17]	Chile	(1) SSB tax revised from 13–18% for SSBs with sugar greater than 6.25 g sugar/100 mL; (2) SSB tax revised from 13 − 10% for SSBs with sugar less than 6.25 g sugar/100 mL	Prices of taxed beverages increased between 2–6.7%	2014
Cuadrado et al. (2020) [97]			The policy reduced the affordability of taxed beverages	2014
Nakamura R et al. (2018) [18]			Tax revision led to a 10–13% reduction in beverage prices	2014
Jensen and Smed (2013) [41]	Denmark	Tax 16 DKK/kg saturated fat (2.14€/kg) on foods with saturated fat above 2.3 g/100 g.	butter prices increased by 8.17–11.38 DKK/kg and margarine prices increased by 4.57–6.18 DKK/kg higher	2011
Jensen et al. (2016) [21]			a 13–16% price increase for high-fat varieties of minced beef and cream products	2011
Etilé F et al. (2018) [98]	France	An excise tax of 0.0716 Euro/Litre on the producer price of SSBs	The pass-through effect of the policy was approximately 39%	2012
Berardi N et al. (2012)			SSB tax was fully shifted to soda and almost fully shifted to the prices of fruit drinks	2012
Capacci et al. (2019) [23]			Full price pass-through to soft drinks	2012
Thow AM et al. (2011) [24]	French Polynesia	No tax for SSBs containing less than 5 g sugar/100 ml: Tax of 20 CFP/L for 5 to 9.99 g sugar/100 ml; 2. The tax of 30 (imp) or 40 (prod) CFP/L for 10 to 29.99 g sugar/100 ml; 3. 45 (imp) or 60 (prod) CFP/L if contains 30 to 39.99 g sugar/100 ml; 4. 60 (imp) or 85 (prod) CFP/L CFP/L if contains 40 g + sugar/100 ml+.	Prices of soft drinks increased by 5.9%	2002
Grogger (2017) [99]	Mexico	An excise tax of one peso ($0.008) per litre	1–2% of mean body mass; price change due to the tax is 1.61 pesos; reduction of 16.7 and 25.4 L per person per year, or 12–18% of the 2013 average	2014
Colchero et al. (2015) [100]			The tax was passed through to all SSBs and overshifted for carbonated SSBs	2014
Aguilera et al. (2017) [51]		8% tax on nonessential energy-dense foods	The snack industry transferred all the tax to the prices of snacks	2014
Salgado and Ng, 2019 [101]			Price increases were larger than the tax rate for only cookies	2014
Gračner et al. (2022) [102]			increased their prices by 4.8% on average	2014

United Kingdom: Scarborough et al. [32] estimated a price increase of £0.075 per litre for high-level drinks, corresponding to a 31% pass-through rate. The price of low-intensity drinks decreased marginally, while that of no-intensity drinks increased marginally. Dickson et al. [34] found that the SDIL was over shifted to soft drink brands that maintained their recipes, leading to a significant increase in their retail prices.

Barbados: Alvarado et al. [96] assessed price changes in SSBs following the implementation of the government’s 10% ad valorem tax. The SSB prices from a major supermarket in Barbados were used for the case study. The authors found that before the tax, both SSBs and non-SSBs had similar year-on-year price growth. However, the growth in SSB prices reached 5.9%, while non-SSB prices grew below 1% after the tax came into effect.

Bermuda: Segal et al. [40] estimated a price increase of 26% for taxed SSB but no impact on the prices of untaxed beverages. In addition, the subsidy policy had no significant impact on the prices of fruits and vegetables sold in the country.

Chile: In Chile, Caro et al. [17] reported that the price of SSBs with a high sugar content increased by 2.0%, while the price of SSBs with a sugar content less than 6.25 g/100 mL decreased by 6.7%. Nakamura et al. [18] found that the purchase prices of soft drinks decreased for items for which the tax rate was reduced from 13 to 10%, but they remained unchanged for sugary items for which the tax was increased. However, they suggest that the purchase prices of SSBs increased when the tax revision was announced. Cuadrado et al. [97] assessed the impact of the tax revision on the affordability of soft drinks and concluded that the policy was effective in increasing prices.

Denmark: Jensen et al. [21] concluded that the Danish fat tax had an insignificant or small negative effect on low- and medium-fat varieties but led to a 13–16% price increase for high-fat varieties of minced beef and cream products. Jensen and Smed [41] assessed the impact of the same policy on butter (8.17–11.38 DKK/kg higher) and margarine (4.57–6.18 DKK/kg higher) and concluded that prices were higher than in the pretax period.

France: Berardi et al. [103] assessed the impact of the French soda tax on prices using French microdata. The authors concluded that the SSB tax was fully shifted to soda and almost fully shifted to the price of fruit drinks six months after implementation. However, the authors found that the pass-through for flavoured water was incomplete. Etilé, Lecocq, and Boizot-Szantai [98] also assessed the impact of French soda taxes on consumer prices and welfare. They showed that the pass-through effect of the policy was approximately 39%, less than that estimated by Berardi et al. [103]. As a result, the prices of SSBs and NCSBs increased by 4% after the tax came into effect. Capacci et al. [23] assessed the impact of the French soda tax and confirmed the findings of Berardi et al. [103], showing that the tax was transmitted to the prices of taxed drinks, with full transmission for soft drinks.

Mexico: Arantxa Colchero et al. [104] assessed the impact of the Mexican excise tax on the prices of SSBs in urban areas. A fixed effect model was applied to data obtained from the National Institute of Statistics and Geography from 2011 to 2014. They found that the tax was passed through to all SSBs and was over shifted for carbonated SSBs. However, the increase in the price of SSBs with small package sizes was greater and differed by region.

Assessing the association between the Mexican tax on nonessential high-calorie foods and consumer prices, Gračner, Kapinos, and Gertler [102] found that the average price of energy-dense food in Mexico increased by 4.8% immediately after the tax came into effect. In addition, price increases were greater in supermarkets than in mini-markets and convenience stores. Grogger [99] also found similar evidence indicating that the price of soda rose by more than the amount of the tax. Aguilera Aburto et al. [51] studied how the prices of snacks changed after the Mexico food and beverage tax by estimating the potential impact of the price increase on the consumption of snacks. Their results indicated that the snack industry transferred all the tax to the prices of snacks. Salgado and Ng [101] found evidence that suggested that price changes might be the result of an increasing price trend rather than tax implementation. In addition, their firm-level analyses mostly showed that price increases by leading firms were greater than the overall increase at the food market level.

Navajo: George et al. [52] reported that the average cost per item of fresh fruit decreased by 13% in Navajo stores but increased by 16% in border stores.

Pacific: Thow et al. [24] reviewed the effectiveness of taxing soft drinks in the Pacific, specifically Fiji, Samoa, Nauru, and French Polynesia. The authors found that, in Fiji, casual monitoring of prices by the Ministry of Health staff suggested that the price of a 2-liter bottle of branded soft drink increased by 10 cents over the first half of 2006 (consistent with a 5-cents/Liter tax increase) from FJ$1.70 to 1.80.

Philippines: Onagan et al. [56] found that the implementation of the sugar-sweetened beverage tax led to a 20.6% and 16.6% increase in the price of sweetened beverages in convenience stores and supermarkets, respectively, a month after the tax came into effect.

Portugal: Gonçalves and Pereira dos Santos [61] reported a full-price pass-through for taxed beverages containing more than 80 g per Liter of sugar and more than a 100% price pass-through for beverages containing less than 80 g per Liter of sugar.

Saudi Arabia and South Africa: Alsukait et al. [63] estimated a pass-through rate of 110% for carbonated drinks after the implementation of the Saudi Arabia SSB tax. Stacey et al. [105] estimated that the price of carbonated drinks increased by 1.006 ZAR/litre following the introduction of the South African SSB tax.

Berkley, USA: Silver et al. [76] assessed the implications of the Berkley beverage tax one year after it came into effect. The results of the study suggested that supermarkets (both large and small) and gas stations had a 100% tax pass-through; pharmacies had a partial tax pass-through, while corner stores and independent gas stations had a negative tax pass-through. Falbe et al. [106] assessed the short-term (3 months after the tax) ability of the Berkely SSB tax to increase retail prices. They found that for smaller beverages (≤ 33.8 oz), the price increases in Berkeley relative to those in comparison cities were 0.47–0.68 cents/oz. For 2-L bottles and multipacks of soda, the relative price increases were 0.46 and 0.49, respectively. However, the prices of nontaxed drinks remained the same. Cawley and Frisvold [107] also assessed the pass-through of the Berkley SSB tax using a difference-in-differences model. They found that across all brands and sizes of products examined, 43.1% of the tax was passed on to consumers.

Boulder, USA: In July 2017, Boulder, Colorado, implemented a two-cents per ounce excise tax on the distribution of beverages with added sugar and other sweeteners. Cawley et al. [108] assessed the pass-through rate of the tax and found that consumers bear most but not all the tax; in both the hand-collected store data and restaurant data, the pass-through was slightly less than 75%, whereas the pass-through was just over 50% using scanner data.

Cook County, USA: Powell and Leider [78] found that prices increased by 1.13 cents per fluid ounce during the 4 months that the Cook County sugar-sweetened beverage tax was implemented. Another study by Powell, Leider, and Léger [109] showed that the tax had a pass-through of 119%, increasing the average price of SSBs by 34%. However, the price increase ranged from a 52% increase for family-size soda to a 10% increase for family-size energy drinks.

Oakland, USA: Marinello, Pipito, et al. [110] used a difference-in-differences analysis to evaluate the effect of the Oakland 1-cent/ounce sugar-sweetened beverage tax on the prices of beverages sold in fast-food restaurants two years after the tax was implemented. The authors found that the price of bottled regular soda increased by 1·44 cents/oz (tax pass-through rate of 144%), and the price of bottled diet soda increased by 1·17 cents/oz. Cawley et al. [111] assessed the impact of the Oakland SSB tax on prices, purchases and consumption by adults and children. They concluded that approximately 60% of the tax was passed on to consumers. Assessing the pass-through effect of the tax two years after its implementation, Leider, Li, and Powell [112] found that taxed beverage prices increased by 0.73 cents/ounce on average in supermarkets and grocery stores in Oakland relative to comparison sites and 0.74 cents/ounce in pharmacies but did not change in convenience stores. Marinello et al. [113] found that the Oakland SSB tax had an 82% pass-through a year after its implementation. They also showed that both diet and regular soda had similar price changes, even though they were not significant.

Seattle, USA: Powell and Leider [82] assessing the impact of the Seattle SBT showed that the prices of taxed beverages increased by 1.04 cents per ounce (59% tax pass-through rate). However, Jones-Smith et al. [114] reported an average increase of 1.58 cents per ounce among Seattle retailers, a pass-through rate of 58–104%. The price increases were greatest for smaller grocery stores and drug stores. Another study by Powell and Leider [84] found a much lower price increase of 1.03 cents per ounce corresponding to a 59% pass-through rate.

Philadelphia, USA: Roberto et al. [86] assessed the impact of the Philadelphia beverage tax on beverage prices and sales in Philadelphia and Baltimore, Maryland (a control city without a tax). The authors found a significant increase in prices: 0.65 cents per ounce at supermarkets, 0.87 cents per ounce at mass merchandise stores, and 1.56 cents per ounce at pharmacies. Bleich et al. [87] found that the Philadelphia beverage tax increased taxed beverage prices by 2.06 cents per ounce, corresponding to a 137% pass-through rate two years after implementation. Cawley, Willage, and Frisvold [115] assessed the pass-through of the tax at the airport and found that prices had increased by 0.83 cents per ounce more in tax than untaxed stores, corresponding to a pass-through of 55.3%. A study by Petimar et al. [91] revealed that taxed beverage prices increased by 1.02 cents per ounce two years after the policy came into effect. Bleich et al. [92] found a much greater impact of the tax, with a 1.81 cents per ounce or a 120.4% increase in prices after the tax was implemented. However, Seiler et al. [93] found that the tax led to only a 34% price increase, corresponding to a 97% pass-through. Cawley et al. [116] found that, on average, distributors and retailers fully passed the Philadelphia SSB tax to consumers. However, the pass-through rate varied by store type, neighbourhood, and proximity to untaxed stores.

### Theme 3: Implication of taxes for health

Table 4 shows a summary of the results discussed in this section. Most of the studies are based on simulating the health implications of implemented government policies. The results conclusively revealed a significant impact of tax policies on improving population health, reducing obesity and related diseases, increasing the number of lives saved, and reducing NCD such as diabetes, ischaemic heart disease and stroke.

**Table 4:** Summary of studies analysing the impact of tax policies on population health

Author	Country	Intervention	Effect on outcomes	Year Implemented
Smed S et al. (2016) [19]	Denmark	Tax 16 DKK/kg saturated fat (2.14€/kg) on foods with saturated fat above 2.3 g/100 g.	123 lives saved annually	2011
Bødker M et al. (2015) [20]			Inconclusive	2011
Cawley et al. (2022) [27]	Mauritius	8 US cents per 100 g of sugar content	No effect on population BMI; Significant effect on BMI of males	2013
Barrientos-Gutierrez et al. (2018) [117]	Mexico	An excise tax of one peso ($0.008) per litre	Average BMI and obesity prevalence reduced	2014
Basto-Abreu et al. (2019) [118]			Reduction in population obesity, and diabetes; and gains in quality-adjusted life-years	2014
Grogger (2017) [99]			Reduction means body mass	2014
Hernández-F et al. (2021) [119]			Lower probability of having dental caries	2014
Saxena et al. (2019) [120]	Philippines	12% value-added tax on SSBs	Reduction in deaths related to diabetes, ischaemic heart disease and stroke.	2018
Goiana-da-Silva et al. (2020) [60]	Portugal	Tax is 1 euro cent per litre for drinks with less than 25 g of sugar per litre, 6 cents for drinks with 25–49.99 g or more sugar per litre, 8 cents for drinks with 50–79.99 g of sugar per litre, and 20 cents for drinks with 80 g or more sugar per litre.	Reduction in BMI and delayed death	2017
Urwannachotima et al. (2020) [121]	Thailand	SSB products that contain less than 6 g sugar per 100 mL are exempt from the tax, while those products containing 6 g or higher sugar per 100 mL are taxed at a higher rate.	Decrease the prevalence of dental caries	2017
Stacey et al. (2019) [105]	South Africa	Tax rate of 2.1c per gram of total sugar in excess of 4 g/100 ml or 10% excise tax	Reduction of Type 2 Diabetes Mellitus related premature deaths	2018
Rogers et. (2023) [122]	United Kingdom	SDIL charges manufacturers and importers at £0.24 per litre for drinks with over 8 g sugar per 100 mL, £0.18 per litre for drinks with 5 to 8 g sugar per 100 mL, and no charge for drinks with less than 5 g sugar per 100 mL.	Decreased prevalence of obesity in year 6 girls, with the greatest differences in those living in the most deprived areas	2018
Rogers et al. (2023) [123]	United Kingdom		Reductions in incidence rates of hospital admissions for carious tooth extraction in children, 22 months post-SDIL implementation	

Rogers et al. [122] assessed the impact of the SDIL on obesity in the United Kingdom and reported that there was a reduction in obesity among 6-year-old girls, with the greatest differences in those living in deprived areas. No significant changes were found for boys. Rogers et al. [123] estimated a relative reduction of 12.1% in hospital admissions for carious tooth extractions in all children (0–18 years) following the implementation of the levy.

Denmark: Smed et al. [19] found that the fat tax imposed on saturated fat saved 123 lives annually, 76 of which were less than 75 years old, equivalent to 0.4% of all deaths from NCD. In general, the tax had a more positive impact on men than women. Bødker et al. [20] also examined the effects of fat tax on the risk of ischemic heart disease (IHD) using retail outlet data on 12 foodstuffs targeted by the tax. The results from the study were inconclusive, suggesting an increase in the population risk of IHD of 0.2%, and the other estimate suggested that the risk of IHD decreased by 0.3%.

Mauritius: Cawley et al. [27] found that the Mauritius SSB tax had no effect on BMI for the full sample of youth considered in their data. However, BMI among male youth was reduced by 11% after the tax was implemented.

Mexico: Using published data on the reductions in beverage purchases due to the Mexican SSB tax, Barrientos-Gutierrez et al. [124] modelled the expected long-term impacts on body mass index (BMI), obesity, and diabetes. Their results showed an average BMI reduction of 0.15 kg/m2 per person, which translates to a 2.54% reduction in obesity incidence. People with the lowest socioeconomic status and those between 20 and 35 years of age had the greatest reductions in BMI and in the prevalence of overweight and obesity. Basto-Abreu et al. [118] assessed the cost-effectiveness of the SSB excise tax in Mexico. The results from their study suggest that the current tax is projected to prevent 239,900 cases of obesity, 39% of which are among children. It could also prevent 61,340 cases of diabetes, lead to gains of 55,300 quality-adjusted life-years, and avert 5,840 disability-adjusted life-years. Grogger [99] concluded that soda price increases could lead to a 2- to 3-point reduction in mean weight, which amounts to approximately 1–2% of the mean body mass. Hernández-F, Cantoral, and Colchero [119] studied the effect of the Mexican food and beverage tax on dental health in Mexico. The authors showed that taxes were associated with a lower probability of having dental caries and with a lower number of teeth with caries experience in the samples studied.

Philippines: Saxena, Koon, et al. [120] modelled the impact of the Philippine’s sweetened beverage tax and reported that the tax could avert an estimated 5,913 deaths related to diabetes, 10,339 deaths from ischaemic heart disease and 7,950 deaths from stroke over 20 years.

Thailand: Urwannachotima et al. [121] assessed the impact of the sugar-sweetened beverage tax on dental caries and concluded that the policy could reduce dental caries in the country by only 1% by 2040.

South Africa: Assessing the impact of the South African HPL on health, Saxena, Stacey, et al. [125] estimated a reduction of 8,000 Type 2 Diabetes Mellitus (T2DM)-related premature deaths over 20 years, with most deaths averted among the third and fourth income quintiles.

Portugal: Goiana-da-Silva et al. [60] estimated that the sugar-sweetened beverage tax prevented 40–78 obese patients per year between 2016 and 2018. Goiana-da-Silva, Cruz-e-Silva, et al. [59] concluded that the decline in sales and SSB consumption due to the tax could translate into 1,600 fewer obese people or delay 27 deaths directly related to excessive sugar consumption in Portugal every year.

### Theme 4: Implications for nontargeted foods

Eleven studies across nine jurisdictions were found to address the impact of taxation on nontargeted foods (See Table 5). The authors found that increasing taxes on unhealthy foods could drive up the consumption of vegetables and water, increase the sales of untaxed food products, and increase the prices of untaxed beverages, juices, etc. It is evident that tax policies have the potential to redistribute consumption towards healthier food options while reducing purchases of unhealthy foods.

**Table 5:** Summary of studies on the impact of taxes on nontargeted foods

Author	Country	Intervention	Effect on outcomes	Year
Smed S et al. (2016) [19]	Denmark	Tax 16 DKK/kg saturated fat (2.14€/kg) on foods with saturated fat above 2.3 g/100 g.	Increased consumption/purchases of vegetables and salt	2011
Capacci et al. (2019) [23]	France	An excise tax of 0.0716 Euro/Litre on the producer price of SSBs	No significant impact on the demand for nontarget foods	2012
Lee MM et al. (2019) [77]	Berkeley, USA	Penny per ounce ($0.01/oz) SSB excise tax	Water consumption increased by 1.02 times per day	2014
Silver, LD et al. (2017) [76]			Sales of untaxed beverages in Berkley increased	2014
Falbe J et al. (2016) [75]			Water consumption increased	2014
Powell et al., (2020) [81]	Cook County	1.00-cent-per-ounce tax on the retail sale of sweetened beverages	No significant change in the volume sold of untaxed beverages	2017
Leider et al. (2021) [112]	Oakland	1 cent per ounce SSB tax on prices	Prices of untaxed beverage increased by 0.40 cents/ounce in pharmacies	2017
Marinello et al. (2020) [113]			Prices of untaxed bottled soda in fast-food restaurants increased	2017
Lalla et al. (2022) [53]	Navajo	Combines a 2% tax on foods of ‘minimal-to-no-nutritional value’ and waiver of 5% sales tax on healthy foods	Water purchasing increased significantly; reduced SSB purchasing	2014
Powell, Leider, and Oddo, (2022) [81]	Seattle	1.75 cents per ounce on distributors of SSBs.	No change in the sales of untaxed beverages	2018
Powell and Leider, (2020) [84]			A 4% increase of volume sold of untaxed beverages	2018
12.Chu et al. (2020) [126]	United Kingdom	SDIL charges manufacturers and importers at £0.24 per litre for drinks with over 8 g sugar per 100 mL, £0.18 per litre for drinks with 5 to 8 g sugar per 100 mL, and no charge for drinks with less than 5 g sugar per 100 mL.	Sugar content of children’s and lunchbox beverages were found to be above the recommended quantities even after the levy implementation	2018

United Kingdom: Chu et al. [126] reported that children’s and lunchbox beverages, though exempted from the SDIL, had higher sugar contents than recommended after the levy implementation.

Denmark: According to Smed et al. [19], the Danish fat tax increased the consumption/purchases of vegetables as well as salt.

France: According to Capacci et al. [23], the 2012 French soda tax did not have any significant impact on the demand for nontargeted foods such as fruit juices and water.

Navajo, USA: Trujillo Lalla et al. [53] reported that the Navajo tax on unhealthy foods and beverages resulted in increased demand for water. Specifically, shoppers in 2019 were 1.5 times more likely to purchase water than were those in 2017.

Berkley, USA: Silver et al. [76] assessed the implications of the Berkley beverage tax one year after it came into effect. They found that the sales of untaxed beverages in Berkley increased. Falbe et al. [75] found that water consumption increased more in Berkley than in the neighbouring untaxed cities of Oakland and San Francisco. A study by Lee et al. [77] concluded that water consumption increased by 1.02 times per day 3 years after implementation.

Cook County, USA: Powell et al. [79] found that the Cook County SSB tax had no significant effect on the volume of untaxed beverages sold in the city or its border area. Marinello et al. [110] reported similar price increases for both taxed and untaxed bottled soda in fast-food restaurants. Leider et al. [112] reported that the price of untaxed beverages increased by 0.40 cents/ounce in pharmacies following the implementation of the tax. However, the price remained unchanged for the other store types.

Seattle, USA: Powell et al. [127] found no change in the sales of untaxed beverages two years after the Seatle SSB tax was implemented. Assessing the implications of the Seattle tax for alcoholic beverages, Powell and Leider [128] reported that the overall volume of alcohol (both beer and wine) sold increased by 4% a year after the tax came into effect and by 5% two years after the tax was implemented. Powell and Leider [84] reported that Seattle SBT had a moderate impact on untaxed beverages, resulting in a 4% increase in volume sold.

Philadelphia, USA: Zhong et al. [85] found a positive impact of the tax on the consumption of bottled water; purchases increased by 58%. However, Bleich et al. (2021b) did not find significant changes in the purchases of nontaxed beverages in Philadelphia. Among high school students, Edmondson et al. [88] found that the tax shifted purchases towards more juice than those in nontaxed cities. Gibson [129] found no evidence of an increase in snacks or spirits following the Philadelphia tax, but there was evidence of substitution for beverage concentrates in supermarkets. Petimar et al. [91] reported that the Philadelphia SSB tax resulted in a 34% increase in the volume of nontaxed beverage concentrates sold; however, there was no evidence of substitution for high-calorie foods. Seiler et al. [93] did not find any significant substitution for bottled water, but there was a modest substitution for untaxed natural juices. Cawley et al. [116], on the other hand, reported that the Philadelphia tax increased the availability of untaxed beverages, particularly bottled water, in Philadelphia stores. Cawley et al. [94] compared the impact of the tax on beverage consumption by children and adults and found that there was no impact on the consumption of other untaxed beverages. Lozano-Rojas and Carlin [130] found that the imposition of the Philadelphia SSB tax increased sugar purchases by 4.3% and 3.7% in neighbouring cities, indicating substitution for other sugary foods. Grummon [95] found that the Philadelphia tax had no impact on other high-calorie/high-sugar nontaxed foods, beverages, or alcohol.

Tonga: Teng et al. [72] and Teng et al. [73] reported a significant increase in bottled water purchases following the implementation of the Tonga sweetened beverage tax.

Spain: Fichera et al. [68] found a very small impact of the Catalonia SSB tax on nontaxed beverages.

South Africa: Stacey et al. [105] showed that the SSB tax had no impact on the prices of nontaxed beverages in South Africa.

### Theme 5: Implications for economic welfare

Seven studies across six jurisdictions were found to assess the impact of taxes on the economic welfare of consumers (see Table 6).

**Table 6:** Summary of studies on the impact of tax policies on economic welfare/distributional effects

Author	Country	Intervention	Effect on outcomes	Year
Etilé et al. (2018) [98]	France	An excise tax of 0.0716 Euro/Litre on the producer price of SSBs	Higher impact on low-income and high-consuming households	2012
Bíró (2015) [25]	Hungary	A 4-cent tax on foods high in salt, sugar or caffeine	Higher impact on lower-income households	2011
Colchero et al. (2017) [45]	Mexico	An excise tax of one peso ($0.008) per litre	Higher impact on lower-income households than all other income groups	2014
Colchero et al. (2017) [31]			Higher among lower-income households, residents living in urban areas, and households with children	2014
Batis et al. (2016) [48]	Mexico	8% tax on nonessential energy-dense foods	Higher impact on lower-income households than higher-income households	2014
Teng et al. (2021) [72]	Tonga	a T$0.50/L sweetened-beverage excise	Higher impact on lower-income households	2013
Phulkerd et al. (2020) [74]	Thailand	SSB products that contain less than 6 g sugar per 100 mL are exempt from the tax, while those products containing 6 g or higher sugar per 100 mL are taxed at a higher rate	High-impact males, older persons, the lower-income population, and the unemployed	2017
Fage and Vasilev (2021) [131]	United Kingdom	SDIL charges manufacturers and importers at £0.24 per litre for drinks with over 8 g sugar per 100 mL, £0.18 per litre for drinks with 5 to 8 g sugar per 100 mL, and no charge for drinks with less than 5 g sugar per 100 mL.	Levy results in a nontrivial welfare loss, particularly in terms of monetary value and weight effect	2018

United Kingdom: A study by Fage [131] concluded that SDIL resulted in nontrivial economic welfare loss, especially among low-income households.

France: Etilé et al. [98]assessing the economic welfare of the French soda taxon, found that the impact of the tax was greater for low-income and high-consuming households.

Hungary: Bíró [25] also assessed the implications of the junk food tax on consumer welfare and found that lower-income households were more affected by the tax.

Mexico: Colcheroet al. [45] found that the Mexican SSB tax had differential impacts on different demographic factors, with greater reductions in SSB purchases for lower-income households, households living in urban areas and households with children. Rivera-Dommarco et al. [31] also confirmed that the Mexican SSB tax affected lower-income households more than all other income groups. In addition, Colchero et al. [46] estimated the impact of the SSB excise tax on purchases of SSBs from stores one year after its implementation and found that lower-income households reduced their purchases more than middle- and higher-income households. Batis et al. [48] also found that the Mexican nonessential energy-dense tax had a greater impact on lower-income households than on higher-income households. Similarly, Hernández et al. [50] found that urban and lower-income households and households with children were more financially affected by the tax on nonessential energy-dense foods. In contrast, Sánchez-Romero et al. [29] did not find any significant variation in the impact of the SSB tax across income levels and consumers based on their educational backgrounds.

Tonga: Teng et al. [72] found that the sweetened-beverage tax had a greater financial impact on low-income than on high-income households in terms of purchase prevalence.

Thailand: Phulkerd et al. [74] showed that the SSB tax in Thailand had a greater impact on males, lower-income populations, older persons and unemployed individuals. Finally, in South Africa, Bercholz et al. [65] showed that the South African SSB tax was more regressive in lower socioeconomic status households.

### Theme 6: Implications for marketing

Table 7 shows the implications of the tax policies for marketing. One study in one jurisdiction analysed the implications of food and beverage taxes for retail marketing. The authors concluded that taxes have a negative impact on retail marketing practices. Oakland: Zenk et al. [132] examined the impact of the Oakland tax on in-store marketing practices—advertising and price promotions. They found that the odds of SSB price promotions fell by 50% in Oakland but only 22% in Sacramento. In addition, price promotions for regular soda declined by 47% at 6 months and 39% at 12 months in Oakland (versus no change in Sacramento). Similarly, the price of artificially sweetened beverages decreased by 55% after 6 months and 53% after 12 months. However, the tax did not affect the advertising of sugar-sweetened or artificially sweetened beverages. Surprisingly, Zenk et al. [133] did not find any long-term (2 years) pre-post impact of the tax on in-store marketing practices—price promotions, exterior or interior advertising, or sale depth—for SSBs and untaxed beverages.

**Table 7:** Summary of studies on other impacts of implemented tax policies

Author	Country	Intervention	Effect on outcomes	Year
Pedraza et al. (2019) [47]	Mexico	Excise tax of one peso ($0.008) per litre	Tax had differential effect across stores	2014
Léger and Powell (2021) [80]	Oakland	1 cent per ounce SSB tax on prices	Evidence of cross-border shopping	2017
Marinello et al. (2021) [134]	San Francisco	1-cent per ounce tax levied on distributors of beverages	No impact on net employment	2018
Powell and Leider (2020) [84]	Seattle	1.75 cents per ounce on distributors of SSBs.	No evidence of cross-border shopping	2018
Chrisinger (2021) [135]	Philadelphia, USA	Beverage excise tax (1.5 cents per ounce)	SNAP shopping in Philadelphia’s neighbouring increased but decreased in Philadelphia	2017
Marinelloet al. (2021) [136]		Beverage excise tax (1.5 cents per ounce)	zero impact of the tax on employment	2017
Saxena et al. (2019) [120]	Philippines	12% value-added tax on SSBs	Estimated total health-care savings of 627 million dollars over 20 years and increase of US$ 813 million in revenue per annum	2018
Gonçalves, dos Santos (2020) [61]	Portugal	Tax is 1 euro cent per litre for drinks with less than 25 g of sugar per litre, 6 cents for drinks with 25–49.99 g or more sugar per litre, 8 cents for drinks with 50–79.99 g of sugar per litre, and 20 cents for drinks with 80 g or more sugar per litre	Stock piling prior to tax implementation	2017
Saxena et al. (2019) [125]	South Africa	Tax rate of 2.1c per gram of total sugar in excess of 4 g/100 ml or 10% excise tax	Government could save US$140 million in subsidised healthcare over 20 years; and would raise US$450 million in tax revenues per annum.	2018
Law et al. (2020) [39]	United Kingdom	SDIL charges manufacturers and importers at £0.24 per litre for drinks with over 8 g sugar per 100 mL, £0.18 per litre for drinks with 5 to 8 g sugar per 100 mL, and no charge for drinks with less than 5 g sugar per 100 mL.	Negative abnormal stock returns on the day of the SDIL announcement which was short lived	2018

### Theme 7: Other impacts

The final scope of the review includes strands of studies that do not fall under themes 1–6. Table 8 shows that these strands of studies assessed the impact of employment in the SSB industry on Supplemental Nutrition Assistance Program Users, stockpiling behaviour, cross-border shopping, government savings and expenditures as well as store type.

**Table 8:** Summary of studies assessing the effectiveness of existing subsidy policies

Author	Country	Intervention	Outcome of Policy	Year Implemented	Conclusion
Galloway (2017) [137]	Canada	Retail subsidy program - The Nutrition North Canada (NNC) Programme	Prices of targeted foods higher than expected	2011	Not achieved
Lu et al. (2016) [138]	Texas, USA	The Special Supplemental Nutrition Program for Women, Infants, and Children (WIC)	The policy improved availability and accessibility of healthy foods	2009	Achieved
Gleason et al. (2011) [139]	Colorado, Pennsylvania, Wisconsin, New Hampshire; USA	WIC program provides supplemental foods, nutrition education, and health and social service referrals to low-income pregnant, nutritional risk.	Increase in the availability of healthier food options	2009	Achieved
Young et al. (2013) [140]	Philadelphia, USA	The Philly Food Bucks - a bonus incentive program at farmers markets	Increase in the consumption of fruits and vegetables	2010–2011	Achieved
Baronberg et al. (2013) [141]	New York, USA	The Health Bucks Programme - a $2 coupon for every $5 spent using SNAP benefits at participating farmers markets	Improvement in sales	2009	Achieved
Black et al., (2013) [142]	Australia	Fruit and vegetable subsidy program: 5 dollars for a box containing 40 dollars’ worth of fruits and vegetables	Fruit and vegetable intake increase; β-cryptoxanthin, vitamin C, and lutein–zeaxanthin levels increased	2005	Achieved
Shamah Levy et al., (2003) [143]	Mexico	Government purchases maize at a given prices and sells it to mills at a lower price.	Reduced cost of Tortilla from 45% of total household food expenditure to 9%	1960s	Achieved

Pedraza et al. [144] found that taxes may have differential effects on different store types; consumers choose different stores to purchase beverages than to purchase foods.

Léger and Powell [80] found cross-border shopping following the implementation of the Oakland SSB tax. However, Powell and Leider [84] found no cross-border shopping associated with Seattle’s sweetened beverage tax.

Marinello et al. [134] assessed the implications of the San Francisco tax on employment and concluded that the tax had no negative impact on net employment, employment in the private sector, or employment in specific SSB-related industries. Marinello et al. [136] also assessed the impact of the Philadelphia beverage tax on employment using synthetic control analysis. The authors found that the city’s employment count was not lower than its synthetic control, indicating that the tax had no impact on employment.

Assessing the Philadelphia SSB tax on the Supplemental Nutrition Assistance Program (SNAP) run by the government, Chrisinger [135] found that the tax contributed to increased SNAP shopping in Philadelphia’s neighbouring counties but decreased spending in Philadelphia.

Gonçalves and Pereira dos Santos [61] found stockpiling by consumers before the implementation of the Portuguese soda tax in 2017. A similar situation occurred in the UK before the implementation of soft drink industry levy. In addition, producers reduce the sugar content of several drinks to pay a lower tax [145].

Saxena et al. [120] found that the Philippine sugar tax could generate total healthcare savings of 627 million United States dollars over 20 years and increase revenue by US$ 813 million annually. Another study by Saxena et al. [125] estimated that the South African government would save US$140 million in subsidised healthcare over 20 years and would raise US$450 million in tax revenues per annum. In Nauru, Thow et al. [24] showed that the government was able to raise approximately US$200,000 because of the tax. In French Polynesia, the total annual revenue increase was US$10 million from domestic production and US$4.2 million from import taxes [24].

### Food subsidy programs

#### The USA

Baronberg et al. [141] assessed the impact of New York City’s Health Bucks Program on EBS at farmers’ markets. In an attempt to increase the accessibility and reduce the cost of fresh produce, health bucks were introduced in 2005 by the New York City Department of Health and Mental Hygiene (DOGHMH). This was a coupon distribution program providing financial incentives for low-income New Yorkers to buy at farmers’ markets in the city’s highest poverty areas. The health bucks were distributed to residents by community-based organisations and could be used at any of the participating markets during the annual growing season (i.e., July 1–November 15). The recipients were SNAP participants who received 2 dollars for every 5 dollars spent using SNAP benefits in participating farmers’ markets. The authors found that farmers’ markets that offered health buck coupons to SNAP recipients had higher average daily EBT sales than markets without incentives. They concluded that implementing the program in other urban areas among low-income shoppers could increase healthful food access and affordability in low-income neighbourhoods.

Young et al. [140] assessed the impact of the Philly Food Bucks program on increasing fruit and vegetable consumption in Philadelphia, USA. From 2010 to 2011, the Food Trust, in collaboration with the Philadelphia Department of Public Health, funded Get Philly to give 2 dollars bonus incentives for every 5 dollars in SNAP that could be redeemed from farmers’ markets only for fresh fruits and vegetables. The goals of the initiative were (1) to increase fruit and vegetable consumption among low-income communities, (2) to increase purchasing power for fruits and vegetables, and (3) to increase the use of SNAP at farmers’ markets. Similar to the program in New York, the coupons were distributed by community-based organisations that served SNAP-eligible populations to promote farmers’ market access among low-income residents. Additionally, the coupons could be redeemed by making a SNAP purchase. The study relied on a convenience sample of 662 customers at 22 farmers’ markets in low-income neighbourhoods in Philadelphia using face-to-face interviews. Their results showed that compared with nonusers, individuals who use food bucks were significantly more likely to report increasing fruit and vegetable consumption. In addition, SNAP sales increased for participating farmers’ markets in low-income communities.

Gleason et al. [139] assessed the impact of the revised WIC Food Package on Small WIC Vendors in four US states. In an attempt to promote healthy diets and reduce childhood obesity epidemics among children and their families in the USA, the Federal Government implemented nutrition programs such as the Special Supplemental Nutrition Program for Women, Infants, and Children (WIC). Participation in the WIC program is limited to low-income pregnant, postpartum, and breastfeeding women and infants and children under the age of 5 years. The impact assessment was based on data collected from WIC-authorised vendors gathered from agencies before and after the package changes were introduced. The authors analysed store inventory data to assess the overall availability of the new WIC foods following the implementation of the new food packages, changes in food availability over time, and how the availability of foods and food categories differed over time by store size and by state. The study revealed that the majority of WIC stores were able to maintain their authorisation status. Additionally, small WIC stores added healthy foods to their inventory in response to the changes in the WIC food package. In addition, the majority of the stores made changes to their registers to meet the new WIC food package requirements. The authors concluded that the implementation of the WIC food package program was generally successful.

In 2016, Lu et al. [138] built on the work of [139] by evaluating the influence of the Revised Special Supplemental Nutrition Program for Women, Infants and Children (WIC) on food allocation packages on healthy food availability, accessibility, and affordability in WIC authorised grocery stores in Texas (a state not included in the Gleason et al. study). They went further to show how the impact of the policy differs among different stores and locations (urban vs. rural). As explained previously, the Special Nutrition Program for Women, Infants, and Children (WIC) was implemented to improve the health of pregnant women and children with low socioeconomic status. The study sampled 105 stores across Texas, and data were collected before and after the implementation of the program. The authors used paired sample t tests to assess the differences before and after the policy implementation. The results from the evaluation study suggest that the availability of most healthy food options (i.e., fruits, vegetables, cereals, and a variety of vegetables) increased in terms of shelf space. The visibility of WIC program labelling increased for fruits, cereals and whole-grain or whole-wheat bread. In general, healthy food availability and visibility increased for stores of different types and in different locations. However, the affordability of healthy foods did not improve in WIC-authorised stores in Texas.

#### Canada

Galloway et al. [146] evaluated the performance of the Nutrition North Canada retail subsidy to ascertain whether it was meeting its goal of making nutritious and perishable food more accessible and affordable in northern counties. Nutrition North Canada was launched by the Aboriginal Affairs and Northern Development Canada (AANDC) in 2011 to offset the cost of transporting perishable foods to northern counties that do not have road access all year round. The program replaced the older Food Mail program, which has offered flight subsidies through Canada Post Corporation since the 1960s. The current program is a federal retail subsidy designed to make nutritious, perishable food more widely available and affordable in northern communities. The author found that there is little evidence to show that the program met its goal of improving the availability of nutritious food. Specifically, the fiscal reporting and food costing tools used by the program were insufficiently detailed to evaluate the accuracy of community subsidy rates and the degree to which retailers are passing on the subsidy to consumers.

In 2017, Galloway [137] performed another comprehensive assessment of the Nutrition North Canada retail subsidy. The assessment was based on program documents, including fiscal and food cost reports for the period 2011 to 2015, retailer compliance reports, audits of the program, and the program performance measurement strategy. The author found that the program lacked a price cap to ensure that food is affordable and equitably priced in communities. In addition, it was difficult to account for the program due to gaps in food cost reporting. The author concluded that the existing structure and regulations of the NNC are not sufficient to ensure that the program meets its goal.

The Mexican government has implemented a subsidy scheme since the mid-1960s. The government has implemented subsidy programs for staple foods usually consumed by poor households. These include maize, wheat, beans, cooking oil, oilseed, rice, sorghum, soybeans and sugar. In principle, the government purchases these foods at the domestic or international market at the prevailing price and then sells them to the processor or packager or directly at a lower price, excluding the distribution and storage cost to consumers. The price consumers pay is set by the Ministry of Commerce. In addition, the government also intervenes in the wholesale and retail of basic foodstuffs. The government’s distribution network reduces the wholesale cost of participating government retail stores and small private shops. Most participating shops are located in low-income urban neighbourhoods. The prices consumers pay in government-run retail shops are estimated to be 10–12% lower than those in nonparticipating stores.

#### UK

The Healthy Start program was introduced in 2006, providing vouchers to pregnant women and families with children younger than 4 years of age who receive certain benefits. Beneficiaries are allowed to exchange vouchers for fruit and vegetables, milk or infant milk. Eligible persons are sent a Healthy Start card containing money for use in retail shops. The card can be used to purchase plain liquid cow’s milk; fresh, frozen, and tinned fruit and vegetables; fresh, dried, and tinned pulses; and infant formula milk. Scantlebury et al. [147] assessed the impact of the Healthy Start program on fruit and vegetable intake among beneficiaries. The authors relied on repeated cross-sectional data from the Healthy Survey for England. Outcomes were compared across the four groups over four time periods: 2001–2003, 2004–2006, 2007–2009 and 2010–2014. This study revealed that during the period from 2001 to 2003 to 2010–2014, fruit and vegetable consumption among adults and children in households deemed eligible for HS changed similarly to that of other adults and children. The authors explained that vouchers might have been spent on other foodstuffs, i.e., milk or infant formula, instead of fruit and vegetables.

#### Mexico

The Mexican tortilla program started in the mid-1960s. The government purchases maize at a given price and sells it to mills at a lower price. The government also absorbs all the distribution and storage costs. The final price of the product, i.e., tortillas, maize flour and maize dough, is set by the government. Assessing the nutritional and economic impact of the tortilla subsidy program, Shamah Levy et al. [143] found that tortilla consumption represented 45% of total household food expenditure and that the subsidy program reduced it to 9%. In addition, the authors found that communities engaged in the program had a lower malnutrition index than those outside of the program.

#### Australia

A fruit and vegetable subsidy program was instituted by the Bulgarr Ngaru Medical Aboriginal Corporation for the Aboriginal Communities in Rural Towns in the Clarence Valley in New South Wales, Australia, in 2005. The beneficiaries paid approximately 5 dollars for a box containing 40 dollars of fruits and vegetables. Low-income households with one or more young children were invited to participate in the program. Black et al. [142] assessed the nutritional impact of the subsidy program and revealed that fruit and vegetable intake increased; β-cryptoxanthin, vitamin C, and lutein–zeaxanthin levels increased significantly after 12 months of participation in the program.

## Discussion

The present study reviewed 127 papers assessing the impact of existing fiscal policies (taxes and subsidies) across the globe on consumer behaviour and the food environment. The studies included in this review were from Europe, Africa, Asia, and South and North America. The results from the various studies were grouped into 7 themes for taxes and 1 theme for subsidies. The themes include the impact of fiscal policies on consumption, purchases, and sales; targeted and nontargeted foods; consumer economic welfare; prices of nontargeted foods; and retail marketing strategies. The studies considered for this review consider different types of taxes and subsidies applied to different types of foods high in fat, sugar and salt. The focus of most fiscal policies is on SSBs or sweetened beverages, foods that are energy-dense and fruits and vegetables. Approximately 39% of the studies are from the United States (comprising states such as Philadelphia, New York, Oakland, San Francisco, Seattle, Navajo, Cook County and Boulder), 16% are from Mexico, 13 are from the United Kingdom, and 4% are from each of the following countries: Chile, Portugal, South Africa, Denmark, France, Hungary, and Spain. Fewer than 4% of the remaining countries evaluated the impact of fiscal measures implemented.

The degree to which fiscal policies can achieve their desired impact is a function of the tax rate and the tax pass-through rate [70]. Most studies suggest a tax rate of 20% and above to achieve significant changes in consumer behaviour. The present review shows two findings: government taxes on SSBs and energy-dense foods are usually less than a 20% price increase, and not all taxes are transmitted to consumers. A lower pass-through rate is usually due to reformulation by firms and the absorption of a significant amount of taxes by manufacturers and retailers to maintain their market shares. Price increases and pass-through rates are different for different countries and even different studies within the same country. For instance, in Philadelphia, Bleich et al. [92] reported a 120% price increase due to the beverage tax; however, Seiler et al. reported a 34% price increase, approximately four times lower than the former. Similarly, in France, Capacci et al. [23]found a full-price pass-through, while Etilé et al. [98] found a 34% price pass-through of the same policy. Silver et al. [76] also showed that a tax policy in one country could have different pass-through rates for different types of stores or shops. This indicates that tax policies are asymmetrically transmitted from point of application to consumers. Despite the profound variations in the results across different jurisdictions, the impact of the policies on prices was positive and significant.

The majority of the studies in this review were centred on Theme 1, the implications of tax policies for consumption, purchases, and sales. A total of 72 studies out of the 126 studies were grouped under this theme. Five out of the 72 studies did not find any impact of the policy on sales, purchases or consumption. However, the majority of the studies found a significant impact of tax policies on the consumption, sales, and purchases of consumers. For instance, studies assessing the implications of the Danish fat tax found that saturated fat purchases fell. A similar result was obtained for Mexico following the implementation of the one peso per Liter excise tax on sweetened beverages. In Chile, although the impact of the policy was found to be small, observable reductions in purchases were confirmed.

The results for the implications of taxes on sales are mixed. Øvrebø et al. [54] and Gibson et al. [129], assessing the implications of government policies for Norway and Philadelphia, found no significant impact on the targeted foods. However, studies from France, Mexico, Hungary, Portugal, Spain, Saudi Arabia, and Berkley (USA) found significant reductions in sales. For instance, Colchero et al. [148] found that SSB sales in Mexico declined by 7.3% per capita sales. In the Philippines, Claire et al. [56] estimated an 8.7% decrease in convenience stores. Castelló and Casasnovas [70] estimated a 7.7% decrease in SSB sales due to the tax. The largest decrease in sales volume was for Saudi Arabia, at 57% from 2010 to 2017. Finally, Goiana-da-Silva, Cruz-e-Silva, et al. [58] estimated a 7% reduction in sales due to the Portuguese sweetened beverage tax. These results show that SSB taxes have a significant impact on retail sales. Studies finding that the positive impact of the tax outweighs those that did not find any impact of taxes and cuts across different jurisdictions.

Various studies have shown that households with lower incomes rely on less nutritious and energy-dense foods for their daily caloric intake [149, 150]. This is evident not only in Scotland but also across different countries and continents. As a result, lower socioeconomic groups suffer financially when fiscal policies are implemented by governments. The results from studies by Etilé F et al. [98], Bíró [25], Colchero MA et al. [31], Batis et al. [151], Teng et al. [72], and Phulkerd et al. [74] confirmed that socioeconomically disadvantaged groups were highly negatively impacted by the tax policies implemented by governments in France, Hungary, Mexico, Tonga and Thailand. In addition, residents, in urban areas, households with children, underemployed individuals, males and older persons or populations are likely to suffer more from tax policies than all other demographic groups. The reason is that these groups derive most of their energy intake from food products imposed with the tax.

Eleven studies assessed the impact of government policies on consumers’ health and nutrition. The evaluation studies were from Denmark (2), Mauritius (1), Mexico (4), the Philippines (1), Portugal (1), Thailand (1) and South Africa (1), which span Europe, Africa, Asia and North America, respectively. Bødker et al. [20] were inconclusive about the implications of the policy in Denmark for health, while Cawley et al. found no effect of the Mauritius policy on the BMI of the average population but a significant effect on the BMI of men. Aside from these two studies, the remaining 9 studies found a significant impact of the policies on population health. Barrientos-Gutierrez et al. [117] and Grogger [99] reported that the average BMI and prevalence of obesity decreased following the implementation of the Mexican sugar-sweetened beverage tax. Saxena et al. [120] estimated a reduction in deaths related to diabetes, ischaemic heart disease and stroke in the Philippines. Urwannachotima et al. [121] and Basto-Abreu, Ana, et al. [118] reported significant reductions in dental caries in Thailand and Mexico, respectively. In Denmark, Smed et al. [19] estimated that 123 lives could be saved annually due to the fat tax. These results confirm the conclusion made by Blakely et al. [152] regarding proposed food taxes and subsidies in New Zealand. In addition, the results of this review support the use of fiscal policies such as all forms of food and nutrition taxes to nudge consumers towards healthy living.

Approximately 24 studies explored the implications of implemented tax policies on nontargeted foods. Seven out of the 24 studies concluded that tax policies had no significant impact on the consumption [94, 129], purchase [23, 79, 95], price [105] or sales [81] of nontargeted food products. The majority of the studies found a significant impact of the policy on nontargeted foods resulting from the substitution effect. For instance, in Tonga, Teng et al. [72] and Teng et al. [73] found a significant increase in bottled water purchases following the imposition and revision of the tax on SSBs. A similar result was found by Lee et al. [77] in Berkley, Lalla et al. [53] in Navajo, and Zhong et al. [85] in Philadelphia. Additionally, in Philadelphia, Edmondson et al. [88] and [93] reported a significant shift in the demand for fruit juice. In Denmark, Smed et al. [19] found that the tax led to a significant increase in the consumption of vegetables. Other authors have found significant increases in the sales [76, 91], purchases [92] and prices [113] of untaxed foods. These results clearly show that fiscal policies tend to have unintended effects on inter-category and intra-category purchases, prices and sales. Therefore, a prior assessment of the tax, as in the case of New Zealand, is relevant before implementation.

The last part of the review on taxes did not fit into any of the proposed themes for this review. However, the results were captured considering their relevance to the topic (see Table). Both [110, 136] found no impact of taxes on employment in the counties (San Francisco and Philadelphia) and the SSB sector. The results from this theme also confirm that the government could raise revenues from taxes. Saxena et al. [120] and Saxena et al. [125] estimated 813 million dollars and 450 million dollars per annum increase in revenue for governments in the Philippines and South Africa, respectively. In addition, both governments could save on healthcare expenditures because of the tax policy. Gonçalves and dos Santos [61] found that the prior announcement of the tax in Portugal resulted in stockpiling. A strategy adopted by consumers to reduce cost or evade price increases due to the tax. Although Léger and Powell [80] found evidence of cross-border shopping in Oakland, Powell and Leider (2020) [84] found no evidence of cross-border shopping in Seattle. As a result, the impact of the policy on cross-border shopping is conclusive. More impact assessments are required to ascertain how consumers along borders react to taxes. Interestingly, the Philadelphia SSB tax had a negative impact on consumers of the Supplemental Nutrition Assistance Program (SNAP).

The final section of the review considered the impact of subsidy policies on sales, purchases and consumption. Four subsidy policies were identified in the US: 1 in Canada, 1 in Australia, and 1 in Mexico. Gleason et al. [139] and [140] reported an increase in the consumption of fruits and vegetables or healthy food options in response to the policy. Baronberg et al. [141] found that subsidising consumers increased the sales of participating farmers’ markets. On the negative side, Galloway (2017) found that the NIP did not have a positive impact on the prices paid by consumers. However, in Mexico, it was found that consumers saved on expenditures on Tortillas.

The current review has the following strengths. First, this is the first study to review studies on existing fiscal policies across the globe. Second, we explored all areas impacted by government fiscal policies irrespective of the jurisdiction and period of implementation. As a result, the review presents governments and policymakers with adequate knowledge on the topic and the types of fiscal policies that have been implemented elsewhere.

A major limitation of the present review is the exclusion of impact studies that are based on simulations and controlled experiments. However, the results from these types of studies may be relevant to the policy they are excluded because they are not based on actual government policies. Another limitation is that we could not ascertain the quality of the papers included in this review, which could impact the conclusions drawn from them. Moreover, the impact of fiscal policies on health is not based on observed changes but rather on expected changes. Future research could compare the results from implemented tax policies with simulation or controlled experimental studies. Finally, a restriction of scoping reviews is that the broad nature tend to ignore quality assessment as a result the quality of the evidence reviewed cannot be confirmed in this study [14].

## Conclusion

Fiscal policies are necessary to make significant changes within the food market environment. This scooping review provides considerable evidence to suggest that existing fiscal policies have improved consumers’ health, increased the prices of targeted food products, increased government revenue, and shifted consumption and purchases towards healthier food options. The impact of fiscal policies is positive for most continents, countries, jurisdictions, consumer groups and store types. Governments could take advantage of fiscal policies to increase revenues, shape consumer attitudes and reduce the burden of diseases and their propounding effects on healthcare costs. There is limited research on the impact of SSB fiscal policies on cross-border shopping and environmental goals.

## Supplementary Information

Supplementary Material 1.

## Data Availability

The datasets used and/or analysed during the current study are available from the corresponding author on reasonable request.

## Footnotes

1 Discretionary foods are foods that are not essential for our health. They are a subset of foods high in saturated fat, sugar and salt comprising confectionery, sweet biscuits, crisps, savoury snacks, cakes, sweet pastries, puddings and sugar containing soft drinks.

2 Ad valorem tax is a tax based on the value of the product; Value-added tax (VAT) is a consumption tax on goods and services that is levied at each stage of the supply chain where value is added; Excise tax is a legislated tax on a product at the time of purchase; and import tariffs are taxes imposed on products imported from other countries.

3 These are foods low in calories, sugar, unhealthy fats, salts, but high in minerals and vitamins.

4 For instance, the Danish fat tax was abolished for financial reasons despite it being effective in the 15 months it was implemented [153, 154].
